# Properties of Alkali-Resistant Glass Fiber Reinforced Coral Aggregate Concrete

**DOI:** 10.3390/ma13163450

**Published:** 2020-08-05

**Authors:** Shutong Yang, Miao Yu, Kun Dong, Yushan Yang

**Affiliations:** 1 Department of Civil Engineering in College of Engineering, Ocean University of China, Qingdao 266100, China; yangshutong@ouc.edu.cn (S.Y.); yumiao1179@stu.ouc.edu.cn (M.Y.); 2 Cooperative Innovation Center of Engineering Construction and Safety in Shandong Blue Economic Zone, Qingdao University of Technology, Qingdao 266033, China; 3 Department of Mathematics and Applied Mathematics, China University of Mining & Technology, Beijing 100083, China; 1710730132@student.cumtb.edu.cn

**Keywords:** coral aggregate concrete (CAC), alkali-resistant glass fiber (ARGF), flexural performance, BFRP bar, bond properties

## Abstract

The intention of this paper is to analyze the properties of coral aggregate concrete (CAC) that is reinforced by alkali-resistant glass fibers (ARGF) and the bond performance with BFRP (basalt fiber reinforced polymer) bars. Two types of ARGF, denoted by Type A and Type B with different manufacturing technologies and fiber lengths, are used in the test. Tests of compressive strength, splitting tensile strength, and flexural performance were performed on ARGF-CAC with four different contents for the two types of ARGF. It is found that the cubic compressive strength is slightly reduced when the fiber volume fraction exceeds 0.5%, but almost keeps invariable if the fiber content further increases. However, the tensile strength, residual strength retention and flexural toughness are improved as more ARGFs are added into CAC, and even higher with Type B ARGF addition. The optimized volume fraction is 1.5% for both the two types of ARGF based on the evaluation of the workability and mechanical performance. Moreover, central pull-out test was performed to study the bond properties of ARGF-CAC with BFRP bars. It is found that both the maximum average bond stress and residual frictional stress are generally reduced as the bond length is longer. The addition of Type B ARGFs can significantly improve the bond strength; however, the Type A ARGFs seem to have marginal effect.

## 1. Introduction

Concrete, as a commonly used construction material, can be widely used in civil engineering. Portland cement, river sands, and crushed stones are essential components in conventional concrete. However, a large amount of CO_2_ emission is inevitable during the cement production [1]. Natural aggregates become scarcer due to the exhaustive exploitation. Researchers attempt to develop replacements of the mentioned ingredients in concrete for the sustainable development of human society. Some minerals having aluminosilicates, such as ground granulated blast furnace slag, fly ash, metakaolin, et al., can be adopted in order to produce alkali-activated materials (AAMs) by using alkali-activators [2]. The Portland cement can be then completely replaced by AAMs in order to mix concrete [3,4]. Besides, some glass waste [5], construction and demolition waste [4,6] can be recycled as fine and coarse aggregates instead of natural aggregates. Eco-friendly green concrete is consequently obtained [4,5]. Moreover, it is well known that the ocean area is much larger than the mainland area on the earth. The latter becomes limited with the rapid development of human society. A great number of marine engineering constructions will be an inevitable trend in the near future and a large amount of concrete would be then utilized. If all the raw materials for concrete mixing are transported by ships from the mainland, the construction cost will be increased and the period becomes longer. Therefore, it is necessary to adopt seawater and sea aggregates instead of the conventional ones in mixing concrete. If so, most of the raw materials can be locally obtained, and the construction cost and period are consequently reduced.

In some islands near tropics, such as South China Sea, there are abundant coral reefs. They can be utilized in a rational manner if no damage is ensured in local ecologies. Subsequently, it is feasible to use the crushed coral reefs as aggregates in concrete production [7]. As the products of many dead coral insects after thousands of years, the main mineral components of coral aggregates are calcium carbonate [8,9], which show considerable difference from those of natural aggregates. The surfaces of coral aggregates are porous and then the aggregates must have high water absorption. Therefore, the coral aggregates are generally pre-soaked in the water before they are mixed in concrete [9]. Because of the high water absorption and irregular shapes of coral aggregates [10], the workability of fresh coral aggregate concrete (CAC) is reduced. However, the early strength develops faster than that of ordinary concrete due to the chloride effects transferred from seawater and coral aggregates [11,12,13,14]. The seven-day strength even exceeds 80% of the 28-day strength [13,14]. Besides, the coral aggregates generally have sufficient bond performance with the surrounding paste and the interfacial transition zones are hardly detected [15,16]. Thus, the fracture of coral aggregates is a common failure mode in CAC [17]. Lyu et al. [18] analyzed the effects of sphericity, angular number and index, shape, and texture of coral particles on the properties of CAC. Incorporations of fly ash, blast furnace slag and metakaolin into CAC can improve the durability of the resulting concrete [19]. As the curing age increases, some of the water in the pre-soaked aggregates would be released and further hydration reaction occurs, which results in subsequent strength improvement [20]. Moreover, because both the seawater and coral aggregates may have high saline content, steel bar cannot be effectively used in reinforced CAC structures. Fiber-reinforced polymer (FRP), as a non-metallic material, can be used instead of steel in CAC structures [13,17,21,22], due to its high resistance to chloride corrosion. Yang et al. [13] analyzed the bond behavior between CAC and FRP bars with different FRP types, diameters, surface types, bond lengths, and curing conditions. Basalt FRP (BFRP) bars have good durability in CAC that is immersed in saline solution [17]. However, Wang et al. [21] found that the bond strength is reduced under the condition of seawater immersion with high temperature. FRP tube can be used as confinement in CAC columns under compression to prevent the spalling failure of CAC [22].

Furthermore, it is found that the failure characteristics of CAC are more brittle than those of ordinary concrete [22,23,24,25]. The ratio of compressive strength to tensile strength is larger and the fracture toughness and energy are lower when compared to ordinary concrete [20]. Therefore, it is necessary to add fibers into concrete to improve both the tensile resistance and toughness [23]. Researchers have attempted to introduce carbon fibers [26], polypropylene fibers [26], sisal fibers [26], and basalt fibers [27,28] into CAC. As the plastic waste is efficiently recycled, Małek et al. [29] successfully added recycled polypropylene plastic fibers in concrete and Kim et al. [30] introduced recycled PET (polyethylene terephthalate) fibers. The results show that the compressive strength, splitting tensile strength, and modulus of rupture are increased with the fiber content, which varies in a certain range [26,27,28,29], and the permeability and shrinkage are reduced [30]. Moreover, glass fibers have been used in civil engineering since the late 1960’s [31]. However, if they are directly added into concrete, the fibers would be corroded under the high alkaline environment. When the surfaces of the fibers are coated with Zirconia having the weight fraction of 15% or more, the alkali-resistance can be improved [31]. Thus, alkali-resistant glass fibers (ARGF) are produced and widely used in construction materials. Incorporating ARGFs can significantly increase the splitting tensile strength [32,33,34,35,36,37], modulus of rupture [32,33,34,35,36,37], flexural toughness [32], and wearing resistance of concrete [33] if no agglomeration of fibers occurs. Crack initiations and propagations are much suppressed, and the maximum crack width and length are reduced [32,35,38]. Proper surface sizing can enhance the environmental resistance of ARGF [31,39]. Besides, the degradation of ARGF in concrete in the aggressive environment is weakened if fly ash and silica fume are incorporated in the concrete [33,40,41]. The resistance to permeability of ARGF reinforced concrete is improved [42].

Although the ARGF is widely used in ordinary concrete, studies on ARGF reinforced CAC are relatively few to our best knowledge. Lu et al. [43] analyzed the cubic strength, splitting tensile strength and resistance to chloride permeability of ARGF-CAC. Only one type of ARGF is considered. In fact, the flexural performance, especially the flexural toughness, should be studied after the addition of ARGFs. Besides, once the ARGF-CAC is used in structures, the bond performance between FRP bars and ARGF-CAC is important and it would dominate the structural behaviors. Thus, the intention of this paper is to study the mechanical properties of ARGF-CAC with different ARGF types and relatively large variation ranges of fiber contents. The bond performance between Basalt FRP (BFRP) bars and ARGF-CAC is then investigated by considering different ARGF types, fiber contents, and bond lengths.

## 2. Experimental Programme

### 2.1. Raw Materials

**Figure 1:**
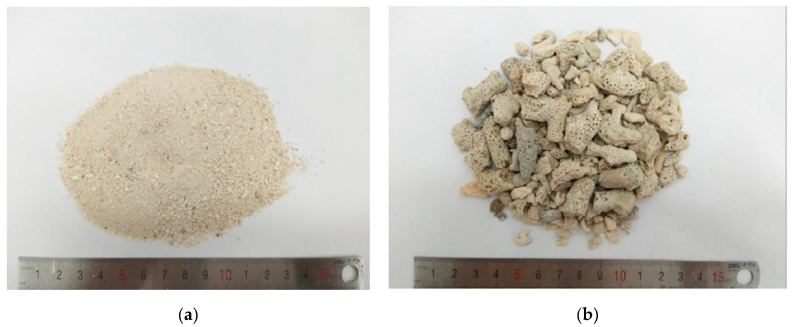
Coral aggregates used in the test: (**a**) Coral sand; and, (**b**) Coral coarse aggregate.

**Figure 2:**
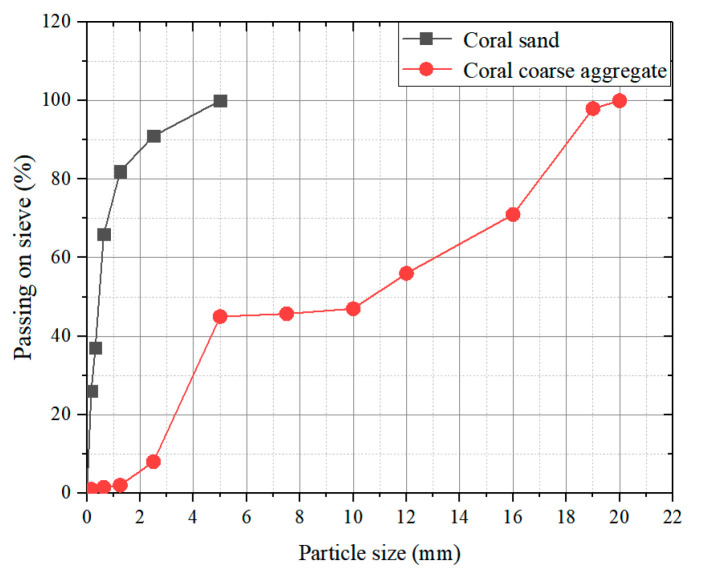
Size distributions of coral aggregates.

In the present study, ARGF reinforced CAC (ARGF-CAC) is mixed by seawater, cement, ground granulated blast furnace slag (GGBFS), Class F fly ash (FA), metakaolin (MK), coral aggregates, and ARGF according to the literature [19]. All of the water used in the test is artificial seawater simulating the water in South China Sea [13]. Ordinary Portland cement (P.O. 42.5) is used as the cement and it is partially replaced by the GGBFS, FA, SF, and MK as the cementing materials. Table 1 provides the main mineral compositions of the latter three admixtures using a X-ray fluorescence (XRF) spectrometer, as per BS EN1926-2 [44]. Figure 1 displays the coral aggregates used in the test. The apparent and bulk densities are 2517 kg/m^3^ and 1236 kg/m^3^ for coral sands, and 1899 kg/m^3^ and 918 kg/m^3^ for coral coarse aggregates (CCA). Coral sands have a continuous gradation of 0–5 mm and coral coarse aggregates have two continuous gradations of 5–10 mm and 10–20 mm, respectively. Figure 2 shows the size distributions.

**Figure 3:**
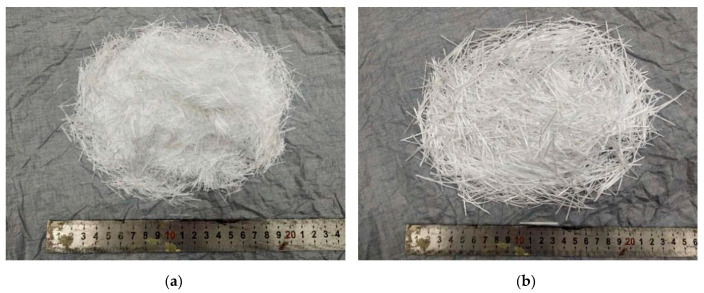
Alkali-resistant glass fibers (ARGF) used in the test: (**a**) Type A ARGF; and (**b**) Type B ARGF.

Two types of ARGF are used in the study and obtained from Tanshan Glass Fiber Limited Company. One of them is Cem-FIL 62 ARGF (denoted by Type A ARGF hereafter) with the length of 18 mm and elastic modulus of 72 GPa. It is bound by 200 fiber filaments with diameters 14 μm. The other one is HP-36 ARGF (denoted by Type B ARGF hereafter), which is bound by 600 fiber filaments with diameters 19 μm. It has the length 36 mm and elastic modulus 72 GPa. The densities of all the ARGFs are 2680 kg/m^3^. The two types of ARGF are seen in Figure 3, as follows. Besides, all of the ARGFs are produced into thin slices and the detailed sizes of the slices are shown in Table 2. Polycarboxylate superplasticizer (PS) with water reducing rate 28% is used in order to improve the workability of fresh concrete.

**Figure 4:**
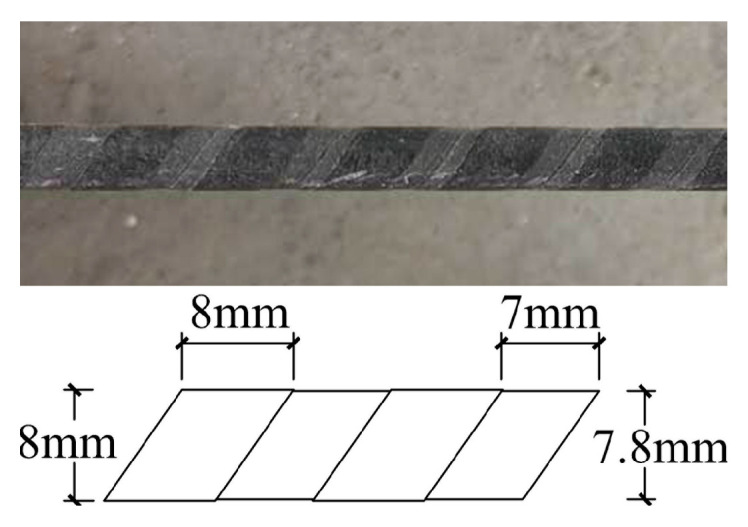
Basalt Fiber-Reinforced Polymer (BFRP) bar used in the study.

BFRP bars are adopted to study the bond performance with ARGF-CAC. All of the bars have nominal diameters 8 mm and shallow ribs on the surface. It should be noted that the ribs are formed by detaching a demoulding belt, which is initially wound on the smooth surface of each bar. The detailed sizes of rib width and spacing for BFRP bar are shown in Figure 4, as follows.

### 2.2. Mix Proportion

It should be noted that the two types of ARGF that are provided by Taishan Fiberglass Inc. have good dispersion properties in the paste. Therefore, relatively large variation ranges of fiber contents are considered in the present study. For each type of ARGF, four different fiber contents, i.e., 0.5%, 1%, 1.5%, and 2%, in the form of volume fractions, are designed. The CAC with no fiber reinforcement is adopted as the control concrete. Moreover, Małek et al. [29] pointed out that the workability of fresh concrete becomes weaker as the fiber content increases. Because the fiber content varies in a relatively wide range in the present study, different superplasticizer additions are needed in order to ensure the slump of all the fresh concrete almost invariable. Thus, the ratios of the superplasticizer to the cementing materials are 0.55%, 0.85%, 1.05%, 1.25%, and 1.55% for fiber volume fractions 0, 0.5%, 1.0%, 1.5%, and 2.0%, respectively. Therefore, five mix proportions of CAC are given in Table 3, as follows, for either Type A or B ARGF.

### 2.3. Mix Production

All of the CCAs were pre-immersed in seawater for 23 h and left in dry condition for 1 h before they were prepared in mixing concrete. SJD-30 mix machine with the maximum range 33 L was used in order to mix the concrete. The rotation rate of the mixer is 45 r/min. During the mixing process, all of the pre-immersed CCAs, coral sands, and cementing materials were first mixed for 30 s in the machine. The seawater and PS were then poured into the solid components. Moreover, the glass fibers are gradually added after the mixture was mixed for 120 s. The fresh concrete is stirred sufficiently with the fiber addition, so that the fibers can be uniformly dispersed in concrete. The whole mixing time lasts about 6 min. The mixing process in the present study is similar to that by Małek et al. [29]. When the fiber volume fraction attains 2%, it is found that some difficulty is encountered more or less during the mixing process. A few fibers are even found to accumulate together. If the fiber content is increased further, the workability of fresh concrete may be too weak to be stirred, and even more PS is added. Segregation, bleeding, and agglomeration of fibers would be then inevitable. Therefore, the maximum fiber volume fraction is allowed to be 2% for both the two types of ARGF.

All of the mix was poured into the moulds for the test specimens, as mentioned in Section 2.5, Section 2.6, Section 2.7, Section 2.8, and compacted for 30 s at a vibration table. The samples were demoulded after 24 h and then cured in a room with temperature 20 ± 2 °C and relative humidity 95%. The tests began after 28 days of curing. 

### 2.4. Test on Slump of Fresh Concrete

The slump values of fresh concrete were determined using a slump cone with the top and base diameters 100 mm and 200 mm, respectively, and height 300 mm, as per BS EN 12350-2 [45].

### 2.5. Determination of Pore Solution pH Value

The dust samples were extracted from the inner regions of the hardened concrete (about 30 mm from the exposed surface), and then dissolved in distilled water, as per RILEM TC 178-TMC recommendations [46]. The pH value is determined while using a pH meter.

### 2.6. Test of Cubic Compressive Strength and Splitting Tensile Strength

**Figure 5:**
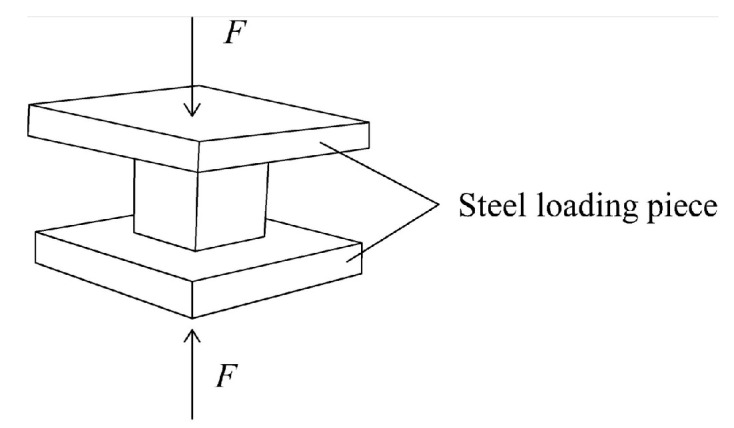
Illustration of cubic compressive strength test.

According to Chinese standard GB/T 50081 [47], three cubic specimens with sizes of 150 × 150 × 150 mm^3^ are adopted in order to determine the cubic compressive strength *f*_cu_ and three cubic specimens with sizes of 150 × 150 × 150 mm^3^ are used to determine splitting tensile strength *f*_ts_. Figure 5 shows the details of cubic compressive strength test with the loading rate 0.6 ± 0.2 MPa/s, as recommended by BS EN 12390-3 [48]. The *f*_cu_ can be expressed by

(1) $$f_{cu}=\frac{F_{u}}{A}$$

where *F_u_* is the maximum applied load at failure and *A* is the cross-sectional area of the specimen.

**Figure 6:**
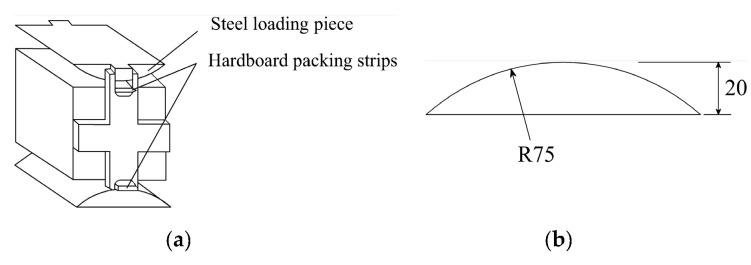
Illustration of splitting tensile strength test: (**a**) Loading configuration; (**b**) Sizes of steel loading piece.

Figure 6 schematically describes the splitting tensile strength test used in the study. The loading rate is 0.05 ± 0.01 MPa/s. When the maximum applied load *F_u_* is reached, the *f_ts_* is given by

(2) $$f_{ts}=\frac{2F_{u}}{\pi A}$$

where the *A* is equal to the square of side length of the cubic sample. 

It should be noted that the specimens in the splitting tensile strength test, as per BS EN 12390-6 [49], are cylinders with diameters 150 mm and heights 300 mm. The test method is very similar to that recommended by Chinese standard GB/T 50081 [47]. However, the A in Equation (2) is equal to the product of the diameter and height of cylinder.

### 2.7. Test on Flexural Performance of ARGF-CAC

**Figure 7:**
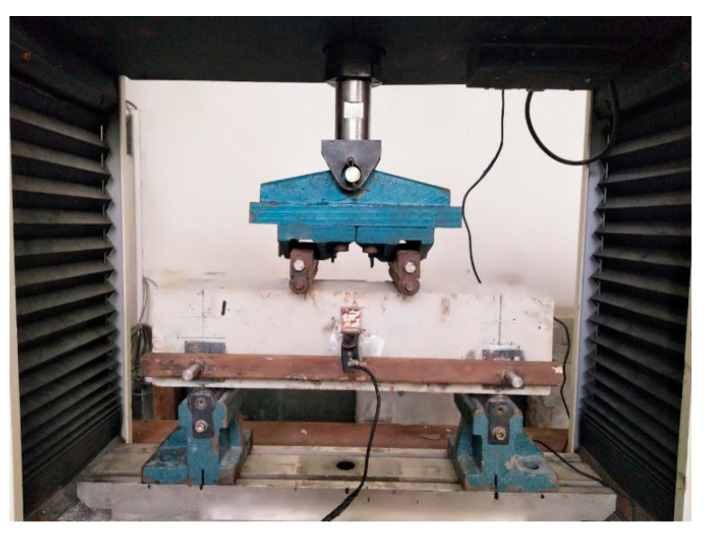
Test set-up for four-point-bending beam.

The test of four-point-bending beams is carried out to evaluate both the flexural tensile strength and toughness of ARGF-CAC according to ASTM C1609/C1609M [50]. The width and depth of beam should be larger than three times of fiber length. Thus, the beams have sizes 100 × 100 × 400 mm^3^ and spans 300 mm for Type A ARGF-CAC. The width, depth, length, and span are 150 mm, 150 mm, 550 mm, and 450 mm, respectively, for Type B ARGF-CAC beams. The bending test is performed on an electronic universal testing machine with the maximum range of 100 kN as shown in Figure 7. Herein, a thin steel plate hangs on the lateral side of beam with two ends fixed at the locations of hinge supports. Thus, it has the same displacement with the two hinge supports when the beam is subjected to the load. Moreover, a steel sheet is bonded on the lateral side of beam at the mid-span. A clip gauge with the maximum range of 4 mm is fixed between the steel sheet and thin plate. Thus, the net displacement of the beam at the mid-span without the effect of hinge support deformation can be directly measured from the clip gauge. The load is applied with the displacement rate 0.2 mm/min. of cross beam in the testing machine. All of the data record from the load and displacement cells can be simultaneously collected in a data acquisition system.

The flexural performance can be comprehensively analyzed by virtue of modulus of rupture *f*_r_, residual strengths $f_{600}^{D}$ and $f_{150}^{D}$ corresponding to net displacements *S*/600 and *S*/150 (*S* is the span length of beam), respectively, beam toughness $T_{150}^{D}$ (the area of the load-displacement curve from 0 to *S*/150), and equivalent flexural strength ratio $R_{T,150}^{D}$, which can be expressed in Equations (3) and (4), as follows [50].

(3) $$R_{T,150}^{D}=\frac{150T_{150}^{D}}{f_{1}bh^{2}}\times 100\%$$

(4) $$f_{1}=\frac{F_{1}S}{bh^{2}}$$

Herein, *b*, *h*, and *F*_1_ are the beam width, beam height, and first peak load, respectively. *f*_1_ is the first-peak strength corresponding to *F*_1_ and equal to *f*_r_ if no first peak load can be detected in the load-displacement curve.

### 2.8. Test on Bond Properties Between BFRP Bars and ARGF-CAC

**Figure 8:**
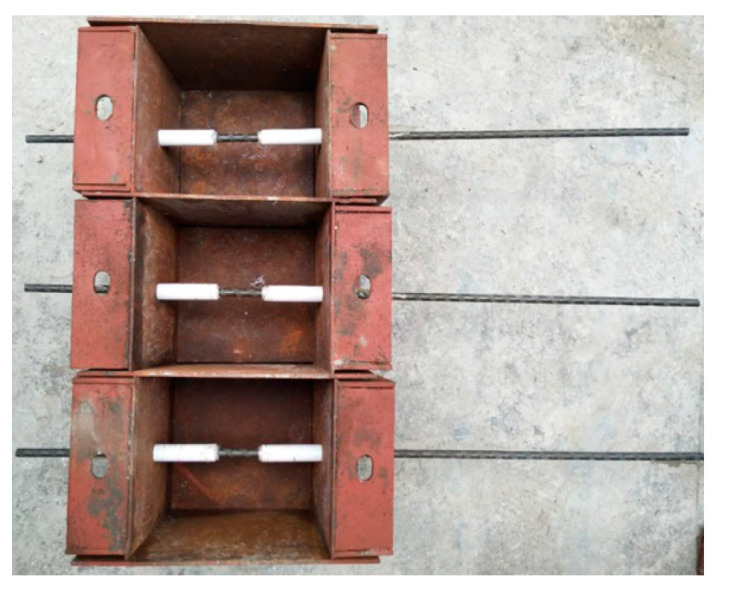
BFRP bars embedded in steel moulds.

Pull-out tests of BFRP bars from ARGF-CAC with sizes of 150 × 150 × 150 mm^3^ are performed in order to study the bond properties between the two materials [51]. Optimized fiber content for the two types of ARGF is determined through evaluating the toughness of ARGF-CAC and adopted to study its influence on the bond behavior. Control specimens of CAC with no ARGF are prepared. There are three main groups of specimens in the pull-out test. In each main group, three bond lengths, i.e., 5*d*, 7.5*d*, and 10*d* (*d* is the diameter of BFRP bar), are designed with five samples for each length. The bond length is controlled by using PVC tubes at two sides of steel mould, as shown in Figure 8.

**Figure 9:**
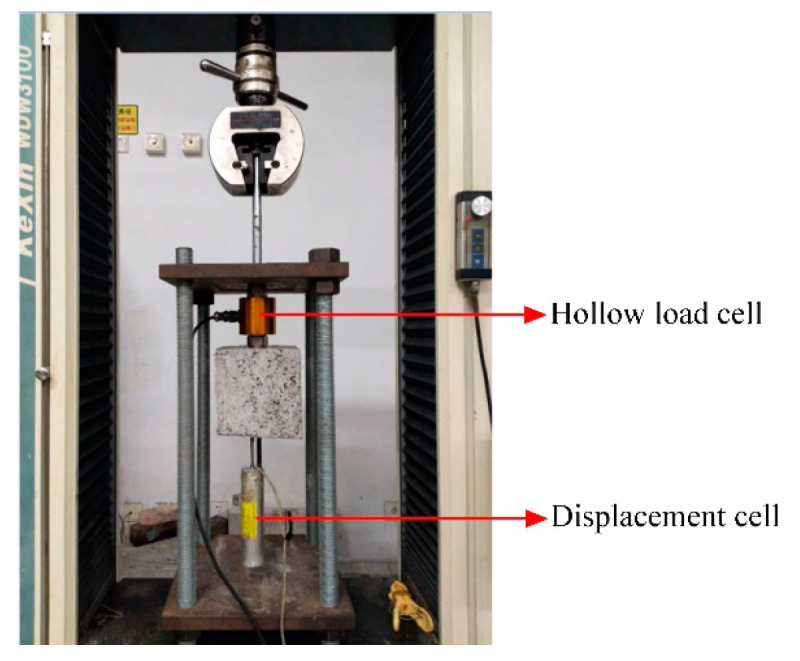
Test set-up for pull-out test.

The electronic universal testing machine with the maximum range of 100 kN is adopted in order to perform pull-out test of BFRP bar from CAC, as shown in Figure 9. Herein, one end of BFRP bar passes through a hollow load cell, is embedded in a steel tube with epoxy and then clipped by the upper grip of testing machine. The load cell is set between the reaction steel plate and upper surface of concrete, and then can monitor the applied load during the pull-out process of BFRP bar. Besides, a displacement cell is tightly attached on the other end of BFRP bar with an extension rod touching on the lower surface of concrete. Thus, the slip between the concrete and BFRP bar at the free end can be measured. All of the data records from the load and displacement cells are simultaneously collected in a data acquisition system.

## 3. Analysis and Discussion on Test Results

### 3.1. Results of Slump Values of Fresh Concrete

The slump values are 115 ± 5 mm for each type of ARGF reinforced concrete. No segregation and bleeding are found in all the fresh mix. 

### 3.2. Pore Solution pH Value

The measured pore solution pH value range is 11.8–12 and the addition of ARGFs has hardly the effect on the pH value.

### 3.3. Results of Cubic Compressive Strength and Splitting Tensile Strength

**Figure 10:**
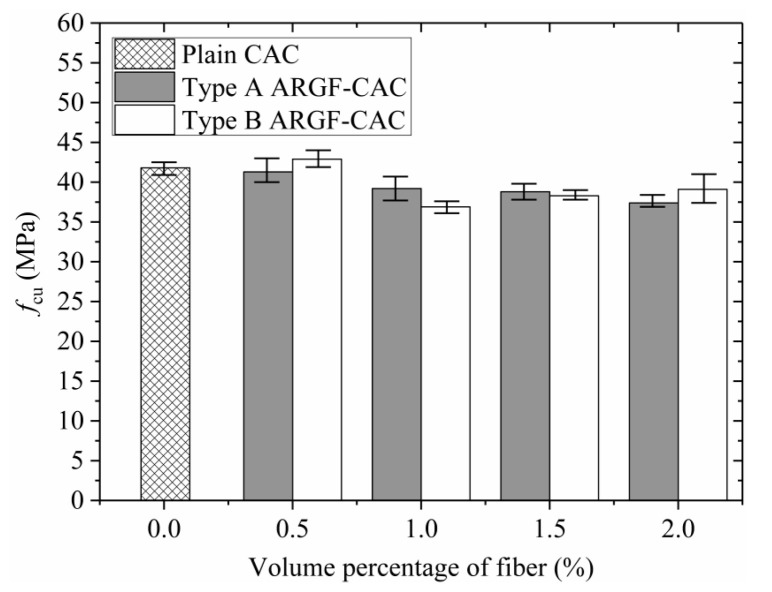
Variation *f*_cu_ with fiber content.

Figure 10 shows the average values of cubic compressive strength *f*_cu_ varying with fiber contents indicating the maximum and minimum values for the two types of ARGF. When the fiber content is below 0.5% by volume, the *f*_cu_ almost keeps invariable for Type A ARGF, but shows a slight increase for Type B ARGF with the increasing of fiber content. However, when the fiber volume fraction exceeds 0.5%, the average values of *f*_cu_ are slightly reduced, as indicated by Sivakumaret al. [36]. As the fiber content further increases, the *f*_cu_ of Type A ARGF-CAC almost keeps constant, but the *f*_cu_ of Type B ARGF-CAC shows a small increment. In fact, the fibers distribute randomly in the concrete and may not be across the longitudinal cracks that are induced by the uni-axial compression. Moreover, even if some of the fibers just distribute across the cracks, the bridging action is so limited due to the insufficient anchorage length when the fibers are very short. If the fiber is long enough, such as Type B ARGF with the length of 36 mm, the bridging effect becomes stronger as the fiber content increases. Thus, the *f*_cu_ is slightly increased when the fiber volume fraction is larger than 1.0% for Type B ARGF-CAC. In summary, the maximum reduction (%) of the average *f*_cu_ is 10.5% and 11.7% for Type A ARGF-CAC and Type B ARGF-CAC, respectively, as compared to plain CAC.

**Figure 11:**
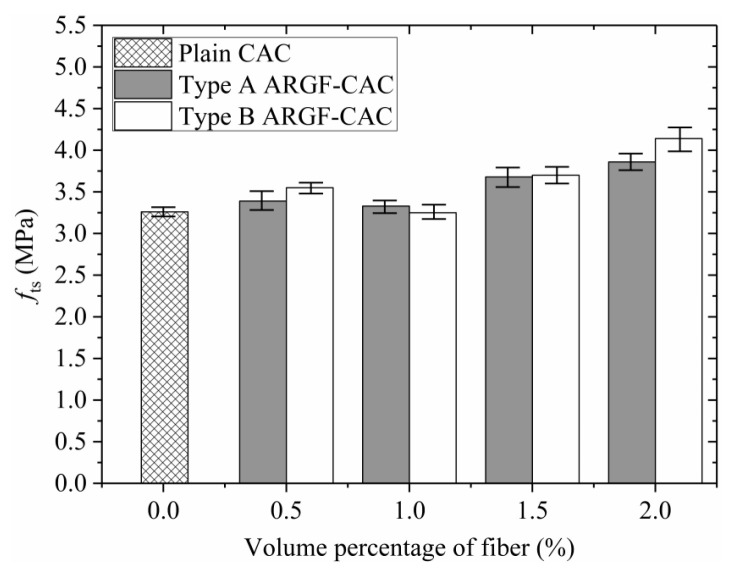
Variation of *f*_ts_ with fiber content.

The average splitting tensile strength *f*_ts_ increases with the fiber content as shown in Figure 11, where the maximum and minimum values in each group are indicated. When the fiber volume fraction is below 1.0%, the *f*_ts_ shows marginal variation with the increasing of fiber content. As the fiber volume fraction further increases, the *f*_ts_ is significantly increased and the maximum increment (%) is 18.4% and 27% for Type A ARGF-CAC and Type B ARGF-CAC, respectively, compared to plain CAC. It demonstrates that both the two types of ARGFs can provide good resistance to crack propagation in the concrete under splitting tension. Moreover, when the fiber volume fractions are the same, the *f*_ts_ is generally improved as the fiber length is longer.

### 3.4. Results of Flexural Performance

**Figure 12:**
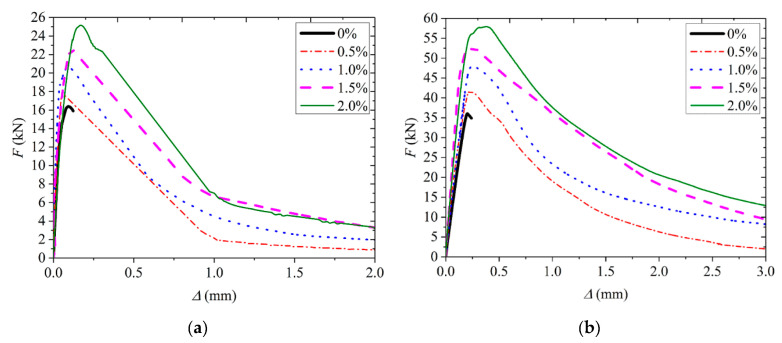
*F*-*Δ* curve: (**a**) Type A ARGF-CAC; and, (**b**) Type B ARGF-CAC.

Figure 12 shows typical curves of applied load *F* varying with the net displacement *Δ* at the mid-span of beam for both the two types of ARGF. It can be seen that the *F* increases almost linearly up to a critical load with the increasing of *Δ* where the cracks initiate from the bottom of beam. A small non-linear portion is observed in each curve until the maximum load is reached. No first peak load is found in all of the specimens. Subsequently, the *F* gradually decreases with the increasing of *Δ* until the beam fails. Moreover, more than 80% of fibers are found to be fractured finally in the critical cross-section, irrespective of fiber type. It means the two types of ARGF used in the study have good bond performance with the CAC.

As the fiber content increases, the peak load is improved due to the stronger bridging effect. Stable descending process can be detected in ARGF-CAC beams. When the fiber volume fraction attains 1.5%, the absolute value of descending slope seems to be the lowest. It demonstrates that the addition of the two types of ARGF can significantly improve the strength and toughness of CAC. However, when the fiber content is larger than 1.5% by volume, the mixing becomes too difficult to be sufficient, as indicated in Section 2.3. Therefore, the descending part of curve is steeper in the CAC beams with ARGF volume fraction 2% than that with ARGF volume fraction 1.5%, as shown in Figure 12.

**Figure 13:**
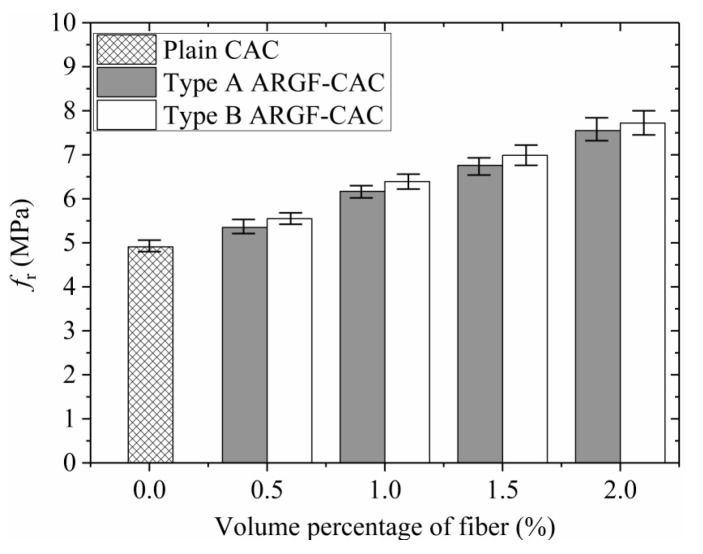
Variation of *f*_r_ with fiber content.

**Figure 14:**
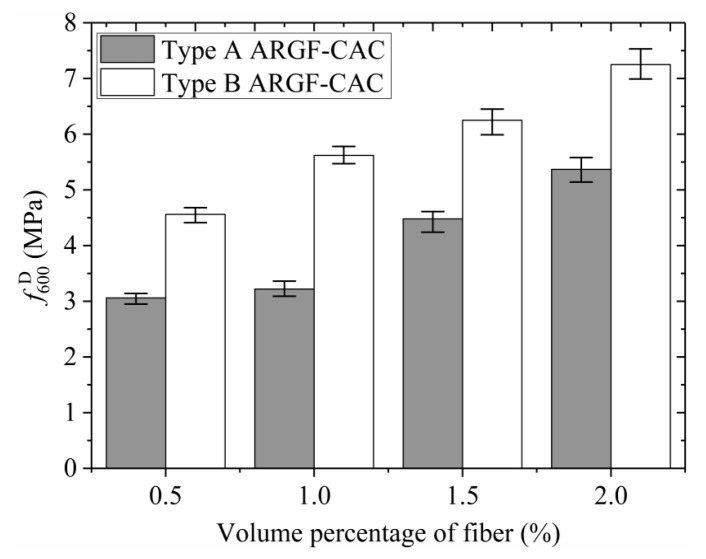
Variation of $f_{600}^{D}$ with fiber content.

**Figure 15:**
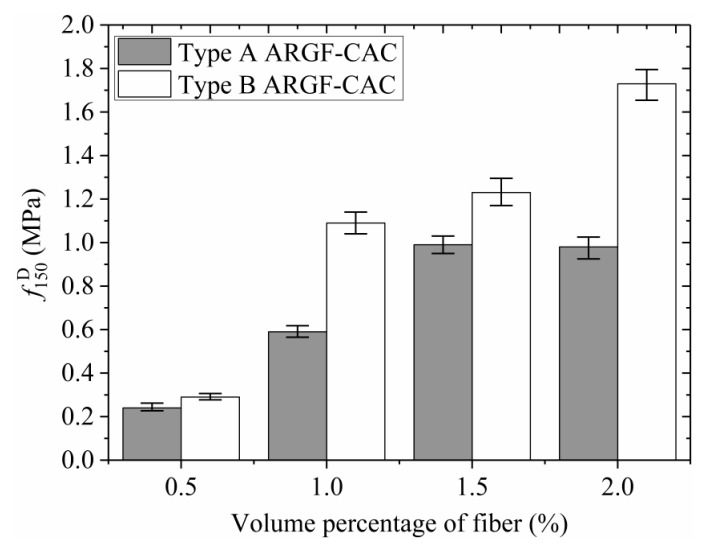
Variation of $f_{150}^{D}$ with fiber content.

**Figure 16:**
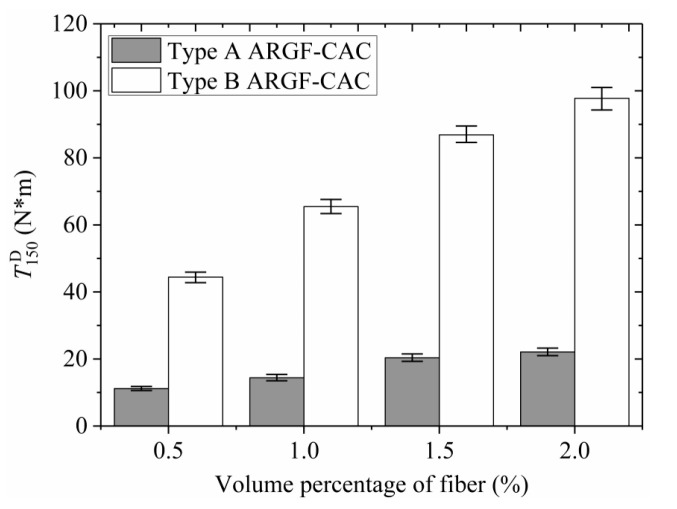
Variation of $T_{150}^{D}$ with fiber content.

**Figure 17:**
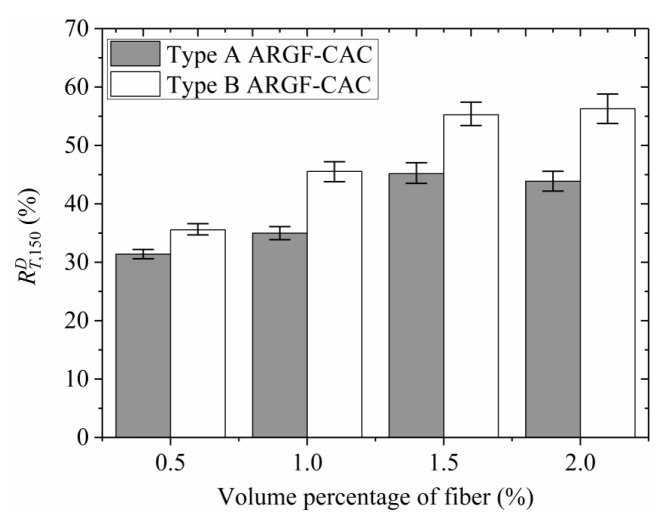
Variation of $R_{T,150}^{D}$ with fiber content.

Moreover, the variations of *f*_r_, $f_{600}^{D}$, $f_{150}^{D}$, $T_{150}^{D}$, and $R_{T,150}^{D}$ with fiber contents indicating the maximum and minimum values are shown in Figure 13, Figure 14, Figure 15, Figure 16 and Figure 17. When the fiber volume fraction is increased from 0.5% to 1.0%, the *f*_r_, $T_{150}^{D}$ and $R_{T,150}^{D}$ have significant increments for each type of ARGF. It again demonstrates that the addition of ARGF can improve both the tensile strength and toughness of CAC. However, if the fiber content increases further, the increases of the mentioned parameters become smaller. When the volume percentage attains 2.0%, the fibers’ distribution in the concrete cannot be as uniform as that in the concrete with fewer fibers, mainly because much difficulty will be encountered in concrete mixing as the fiber content increases. Therefore, the improvement of tensile strength and toughness is very limited if the volume fraction is increased from 1.5% to 2.0%. For Type A ARGF-CAC, it can be seen that the $f_{600}^{D}/f_{r}$ are 57.2%, 52.2%, 66.3%, and 71.1% for fiber volume fractions 0.5%, 1.0%, 1.5%, and 2.0%, respectively. The $f_{150}^{D}/f_{r}$ are 4.5%, 9.6%, 14.6%, and 13% for the four fiber contents, respectively. For Type B ARGF-CAC, the values of $f_{600}^{D}/f_{r}$ and $f_{150}^{D}/f_{r}$ corresponding to the four fiber volume fractions are 82.2%, 87.9%, 89.4%, 93.9% and 5.2%, 17.1%, 17.6%, 22.4%, respectively. The addition of ARGF into CAC results in high residual strength retention, which increases with the increasing of fiber content and length.

According to the above analysis in the present study, the optimized fiber volume fraction for the two types of ARGFs should be 1.5% by considering actual mixing process of fibers in fresh concrete. Thus, the following section will be aimed at the bond performance between BFRP bars and ARGF-CAC with fiber volume fractions 1.5%. Moreover, the specimens of BFRP bars that are bonded to plain CAC are used for the control ones.

### 3.5. Bond Properties Between BFRP Bars and ARGF-CAC

**Figure 18:**
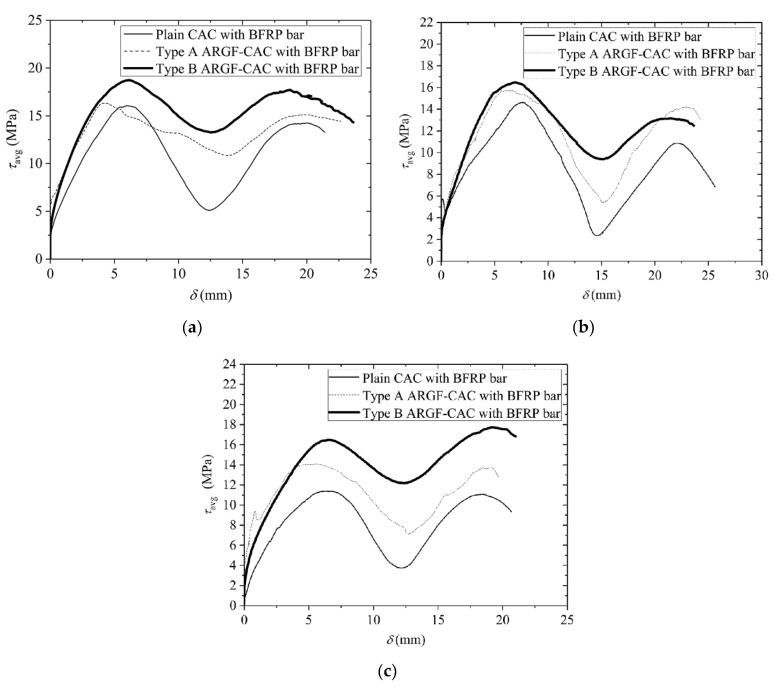
*τ*_avg_-*δ* curves for BFRP bar: (**a**) *L* = 5*d*; (**b**) *L* = 7.5*d*; and, (**c**) *L* = 10*d.*

As discussed above, the present study is only aimed at the bond properties between BFRP bars and ARGF-CAC with volume fractions 1.5%. Control specimens are prepared for the bond behavior of BFRP bars with plain CAC. The intention of this section is to compare the pull-out behaviors of BFRP bars from CAC with different fiber types and different bond lengths. However, it should be noted that the actual bond length *L* may be varied, even for the same nominal bond length (5*d*, 7.5*d*, or 10*d*). Thus, the average bond stress *τ*_avg_ determined by Equation (5) is introduced for the sake of comparison. Figure 18 summarizes the typical *τ*_avg_-*δ* curves under different conditions. Moreover, the specimens have the failure modes of BFRP bar pull-out from the concrete in general. Only three specimens of plain CAC bonded with BFRP bars having nominal bond lengths 10*d* fail by concrete fracturing before the bars are pulled out.

(5) $$\tau_{avg}=\frac{F}{\pi dL}$$

Very similar to the pull-out behavior of FRP bar from CAC [13] and alkali-activated slag seawater sea sand concrete [52], the *τ*_avg_-*δ* curve of each specimen still includes four stages. At the beginning of loading, the *F* or *τ*_avg_ increases with no slip until the frictional stress *τ*_s_ is exceeded. Subsequently, the *τ*_avg_ is further increased with the increasing of *δ*, but the ascending slope becomes lower. When the maximum value is reached, the applied load is gradually reduced, but the *δ* still increases. The final part of each curve is a fluctuation line that is also verified in the bond behavior between FRP bar and alkali-activated slag seawater sea sand concrete [52]. In fact, the bond action almost disappears in the stage and the resistance is mainly provided by interfacial residual friction. The residual frictional stress *τ*_s_ is then adopted as the average value between the maximum and minimum values at the frictional stage.

**Figure 19:**
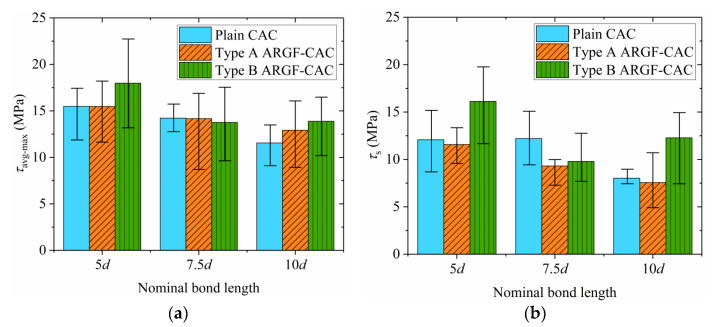
Effects of nominal bond length: (**a**) *τ*_avg-max_; (**b**) *τ*_s._

**Figure 20:**
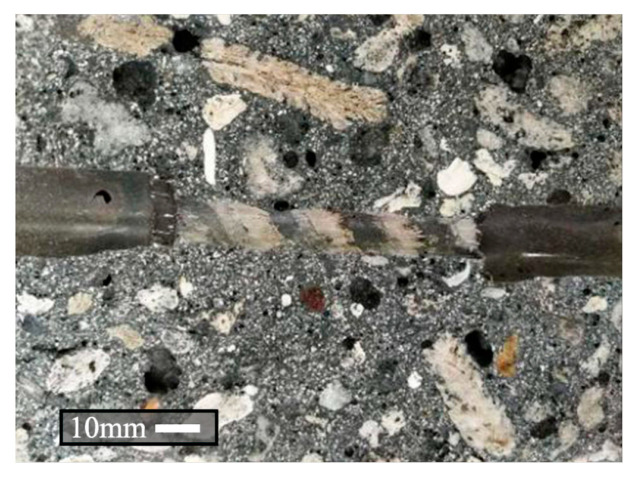
Detached ribs on the surface of fiber-reinforced polymer (FRP) bar.

The variations of maximum average bond stress *τ*_avg-max_ and residual frictional stress *τ*_s_ with the nominal bond length being shown in Figure 19. The maximum and minimum values of the *τ*_avg-max_ and *τ*_s_ in each group are also included in Figure 19. As the nominal bond length increases, the average values of *τ*_avg-max_ and *τ*_s_ commonly decrease. The reasons can be explained, as follows. First, it is acceptable that the non-uniformity of interfacial stress distribution becomes more significant as the bond length increases. It then results in the reductions of the two parameters with the increasing of *L*. Second, the BFRP bars used in the test have shallow ribs on the surfaces. As the bar is pulled out from the concrete, the ribs are seriously detached from the BFRP surface, as shown in Figure 20. The detached ribs may give local compression with surrounding concrete. The local action is stronger as the bond length is shorter, as indicated by Yang et al. [52]. Thus, the average values of *τ*_avg-max_ and *τ*_s_ for specimens with *L* = 5*d* are larger. Besides, radial compression on the bar-to-concrete interface provided by the ribs is inevitable. It may be so strong that the concrete would be cracked along the radial direction. The longer the bond length is, the larger the crack opens. The concrete would be completely fractured before the bar is pulled out if the *L* is long enough, such as some of the specimens with nominal bond lengths 10*d*. The *τ*_avg-max_ and *τ*_s_ are reduced as the radial cracks develop in the concrete. Moreover, the values of *τ*_s_ from different specimens are relatively scattered, because the detachment levels of BFRP surfaces are different along the embedment length.

When the ARGFs are added into the CAC, the *τ*_avg-max_ shows a certain increment. It means that the ARGFs can provide good bridging action on the crack development in the concrete. However, for Type A ARGF, the increase is marginal. If the Type B ARGF is used, the *τ*_avg-max_ is significantly improved. When the *L* is increased from 7.5*d* to 10*d*, the reduction of *τ*_avg-max_ is apparently lower for ARGF-CAC. It demonstrates again that the development of cracks in the concrete is well restricted with the addition of ARGFs. Moreover, the Type A ARGF has a marginal effect on the *τ*_s_, but the incorporation of Type B ARGFs apparently increases the *τ*_s_.

## 4. Conclusions

The present study is mainly aimed at the mechanical properties of ARGF reinforced CAC. Two types of ARGF, denoted by Type A and Type B, are used here. Tests of basic mechanical properties and flexural performance are carried out on both the plain CAC and ARGF-CAC with four contents by volume, i.e., 0.5%, 1.0%, 1.5%, and 2.0%, for the two types of ARGF. The optimized fiber volume fractions for both types of ARGF are determined based on evaluating both the workability and mechanical properties of ARGF-CAC. Moreover, a central pull-out test is performed in order to study the bond performance between BFRP bars and both the plain CAC and ARGF-CAC with the optimized fiber volume fraction. Three nominal bond lengths (5*d*, 7.5*d*, and 10*d*) are considered for each type of concrete. The main conclusions are then drawn, as follows.

(1) As the fiber content increases, the workability of fresh concrete becomes weaker. The average value of *f*_cu_ increases as the fiber volume fraction is increased to 0.5%, especially for Type B ARGF. When the fiber volume fraction exceeds 0.5%, however, the average values of *f*_cu_ are slightly reduced for either Type A or Type B ARGF. As the fiber content further increases, the *f*_cu_ of Type A ARGF-CAC almost keeps constant, but the *f*_cu_ of Type B ARGF-CAC shows a small increment. But the *f*_ts_ and *f*_r_ are generally improved with the increasing of fiber volume fraction.
(2) Both the residual strength retention and flexural toughness are improved, as more ARGFs are added into CAC, and higher with Type B ARGF addition when compared to Type A ARGF. The improvement becomes smaller when the volume fraction of ARGF exceeds 1.0% for either Type A or Type B ARGF. The optimized volume fraction is 1.5% for both the two types of ARGF by considering the workability and mechanical performance of ARGF-CAC.
(3) More than 90% of the specimens have the failure modes of BFRP bar pull-out from the concrete. Only three specimens of plain CAC bonded with BFRP bars having nominal bond lengths 10*d* are damaged by concrete fracturing before the bars are pulled out. When the bar is pulled out, the ribs on the surface are seriously detached.
(4) Both the *τ*_avg-max_ and *τ*_s_ are reduced if *L* > 5*d*. The values of *τ*_s_ show relatively large scatters due to the random detachment of ribs on the BFRP surface. When the Type B ARGFs are added into the CAC, the *τ*_avg-max_ is increased significantly. However, the Type A ARGFs seem to have marginal effect on the bond performance.

## Figures and Tables

**Table 1:** Mineral compositions of admixtures (%).

Type	CaO	SiO_2_	Al_2_O_3_	MgO	SO_3_	TiO_2_	Fe_2_O_3_	K_2_O	Na_2_O
FA	8.4	41.7	34.2	1.15	1.51	1.63	5.92	0.89	3.26
GGBFS	38.3	30.3	16.04	8.26	2.75	2.22	0.63	0.449	0.406
MK	0.12	54.7	40.2	0.084	0	0	0.84	0	0

**Table 2:** Sizes of ARGF slices.

Type of ARGF	Average Thickness (mm)	Average Width (mm)	Length (mm)
Type A	0.12	0.54	18
Type B	0.22	1.08	36

**Table 3:** Mix proportion (kg/m^3^).

Mix Symbol	Seawater	Cement	FA	GGBFS	MK	CS	CCA	PS	ARGF
Base mix	190	300	75	75	50	556	712	2.75	0
Type A ARGF-0.5								4.25	13.4
Type A ARGF-1.0								5.25	26.8
Type A ARGF-1.5								6.25	40.2
Type A ARGF-2.0								7.75	53.6
Type B ARGF-0.5								4.25	13.4
Type B ARGF-1.0								5.25	26.8
Type B ARGF-1.5								6.25	40.2
Type B ARGF-2.0								7.75	53.6
